# Integrated analysis of RNA-binding proteins in human colorectal cancer

**DOI:** 10.1186/s12957-020-01995-5

**Published:** 2020-08-22

**Authors:** Xuehui Fan, Lili Liu, Yue Shi, Fanghan Guo, Haining Wang, Xiuli Zhao, Di Zhong, Guozhong Li

**Affiliations:** Department of Neurology, The First Affiliated Hospital of Harbin Medical University, 23 You Zheng Street, Harbin, 150001 Heilongjiang Province People’s Republic of China

**Keywords:** Colorectal cancer (CRC), RNA-binding protein (RBP), Prognostic model construction, Survival analysis

## Abstract

**Background:**

Although RNA-binding proteins play an essential role in a variety of different tumours, there are still limited efforts made to systematically analyse the role of RNA-binding proteins (RBPs) in the survival of colorectal cancer (CRC) patients.

**Methods:**

Analysis of CRC transcriptome data collected from the TCGA database was conducted, and RBPs were extracted from CRC. R software was applied to analyse the differentially expressed genes (DEGs) of RBPs. To identify related pathways and perform functional annotation of RBP DEGs, Gene Ontology (GO) function and Kyoto Encyclopedia of Genes and Genomes (KEGG) pathway enrichment analyses were carried out using the database for annotation, visualization and integrated discovery. Protein-protein interactions (PPIs) of these DEGs were analysed based on the Search Tool for the Retrieval of Interacting Genes (STRING) database and visualized by Cytoscape software. Based on the Cox regression analysis of the prognostic value of RBPs (from the PPI network) with survival time, the RBPs related to survival were identified, and a prognostic model was constructed. To verify the model, the data stored in the TCGA database were designated as the training set, while the chip data obtained from the GEO database were treated as the test set. Then, both survival analysis and ROC curve verification were conducted. Finally, the risk curves and nomograms of the two groups were generated to predict the survival period.

**Results:**

Among RBP DEGs, 314 genes were upregulated while 155 were downregulated, of which twelve RBPs (NOP14, MRPS23, MAK16, TDRD6, POP1, TDRD5, TDRD7, PPARGC1A, LIN28B, CELF4, LRRFIP2, MSI2) with prognostic value were obtained.

**Conclusions:**

The twelve identified genes may be promising predictors of CRC and play an essential role in the pathogenesis of CRC. However, further investigation of the underlying mechanism is needed.

## Introduction

As a significant class of cellular proteins, RNA-binding proteins (RBPs) can interact with RNA by recognizing special RNA-binding domains and are widely involved in multiple posttranscriptional regulatory processes, such as RNA shearing, transport, sequence editing, intracellular localization and translation control [1]. It is estimated that there are up to 1500 different proteins that have the potential to bind RNA in the human genome [2]. RBPs are characterized by the presence of an RNA-binding domain (RBD) that contains 60–100 residues and usually adopts an αβ topology. Found in single or multiple copies, these domains usually bind to RNA depending on the exact sequence or structure [3]. To date, RBPs have been reported to be associated with various human diseases, such as spinal muscular atrophy and myotonic dystrophy [4]. There are various RBPs involved in tumourigenesis. SRC associated with 68 kDa mitosis (SAM68) is a member of the STAR (signal transduction and RNA metabolism activation) family of RBPs. It is involved in several steps of mRNA metabolism, such as transcription, alternative splicing and nuclear export. In addition, SAM68 is associated with the signal transduction pathways required for the response of cells to stimuli, cell cycle transition and viral infection [5]. TARBP2 is overexpressed in metastatic cells and metastatic human breast tumours, and its abnormal activation can promote the progression of breast carcinomas by affecting the stability of its target mRNA [6].

Colorectal cancer (CRC), which includes colon and rectal cancer, is a common digestive tract tumour. The molecular pathogenesis of CRC is a complex multistep process involving multiple acquired genetic and epigenetic abnormalities [7]. Some RBPs are known to be associated with colorectal cancer. According to some studies, muscleblind-like 1 (MBNL1), an RBP implicated in developmental control, can significantly suppress CRC cell metastasis in vitro. MBNL1 destabilizes snail transcripts and thus inhibits the epithelial-mesenchymal transition (EMT) of CRC cells through the snail/E-cadherin axis in vitro. RAS oncogene activation mutations are commonly seen in colon cancer [8].

In this study, an analysis was conducted of RBP-related genes in CRC patients through differential gene expression and protein molecule interactions. In addition, a prognostic model was adopted to identify twelve genes associated with the survival of CRC patients. We verified the model and performed survival analysis and risk assessment. These results will help elucidate the underlying mechanism related to the survival of CRC at the molecular level, thus providing a new direction for the prognosis of CRC and clinical treatment.

## Methods

### Data source

**Fig. 1:**
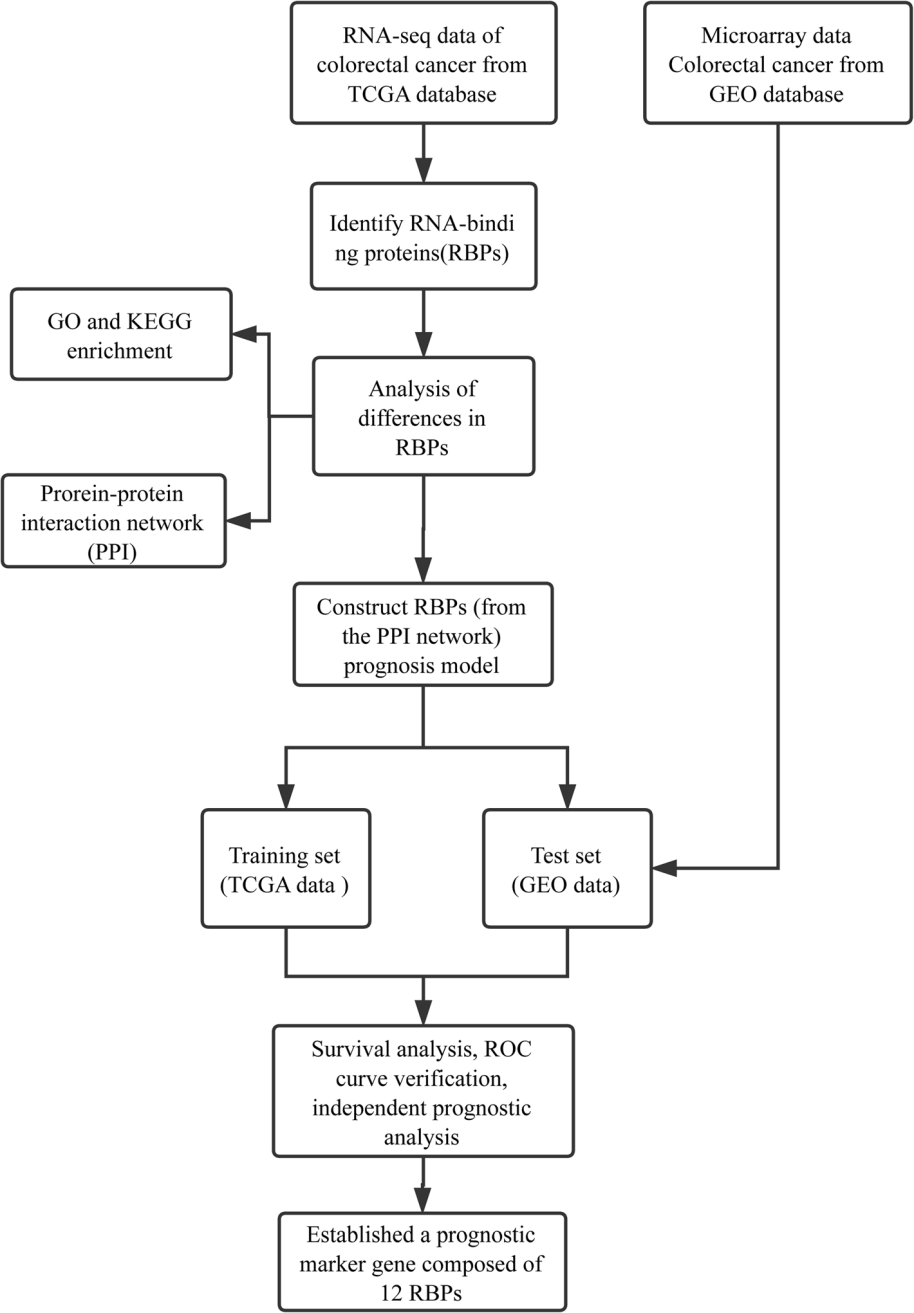
Flowchart of systemic analysis of RNA-binding protein in patients with CRC

The FPKM transcriptome data of CRC were obtained from the TCGA database website (https://portal.gdc.cancer.gov/). The total number of samples is 521, of which there are 479 samples in the tumour group and 42 samples in the normal group. Then, the RBP gene was obtained from the GOA database website (https://www.ebi.ac.uk/GOA/). Combined with the CRC transcriptome sequencing map, CRC RBPs were obtained. The data on gene expression (GSE17536) in colorectal patients were obtained from the GEO database website (https://www.ncbi.nlm.nih.gov/geo/), involving a total of 177 cases. All the data were publicly available online. This study requires no experiments to be conducted by any author on humans or animals. The flowchart of it is shown in Fig. 1.

### Data processing of differentially expressed genes (DEGs)

The RBPs were analysed using R software to identify the difference between the tumour group and the sample group. Wilcoxon test was carried out to identify DEGs between the two groups, with the adjusted *P* < 0.05 and |logFC| > 0.5

### GO and KEGG pathway analysis of DEGs

GO analysis represents a common method applied to conduct large-scale functional enrichment study. Gene functions can be categorized into biological processes (BP), molecular functions (MF) and cellular components (CC). KEGG is known as a commonly used database where a large amount of data on genomes, biological pathways, diseases, chemicals and drugs is stored. Through GO and KEGG analysis of DEGs, barplot and bubble were drawn respectively. All of the GO and pathway terms were ranked by their −log10 (*q* value).

### Protein-protein interaction (PPI) network

The Search Tool for the Retrieval of Interacting Genes (STRING) database (https://string-db.org/) is designed to analyse the PPI information. DEGs were input into the STRING database to obtain PPI information. Subsequently, the Cytoscape software was applied to visualize the PPI network, the Cytoscape plug-in MCODE was used to obtain the most relevant sub-network module and then the hub genes of the four modules were enriched for GO and KEGG analysis.

### Construction and analysis of prognostic models

Cox regression analysis was conducted on the prognostic value of 442 RBPs (from the PPI network) with survival time, the RBPs related to survival were identified and a forest map was generated. Then, the samples of the TCGA database were designated as the training set, and the samples of the GEO database were treated as the test set to construct the best prognostic model based on the training set. Twelve survival-related genes were identified by the model, based on which the correlation coefficient of each gene was obtained. Then, the risk score of each patient in the training set and test set was calculated according to gene expression. In addition, the patients were classified into high-risk and low-risk groups by the median value of the risk score. The patients in the training set and the test set were categorized into either the high-risk group or low-risk group. A survival analysis was conducted, an ROC curve was generated and then the risk curves were constructed for the training and test sets. Furthermore, with univariate and multivariate analyses, nomograms based on the genes obtained from the prognostic model were generated to predict the length of survival for the patients.

## Results

### Identification of RBPs DEGs

**Fig. 2:**
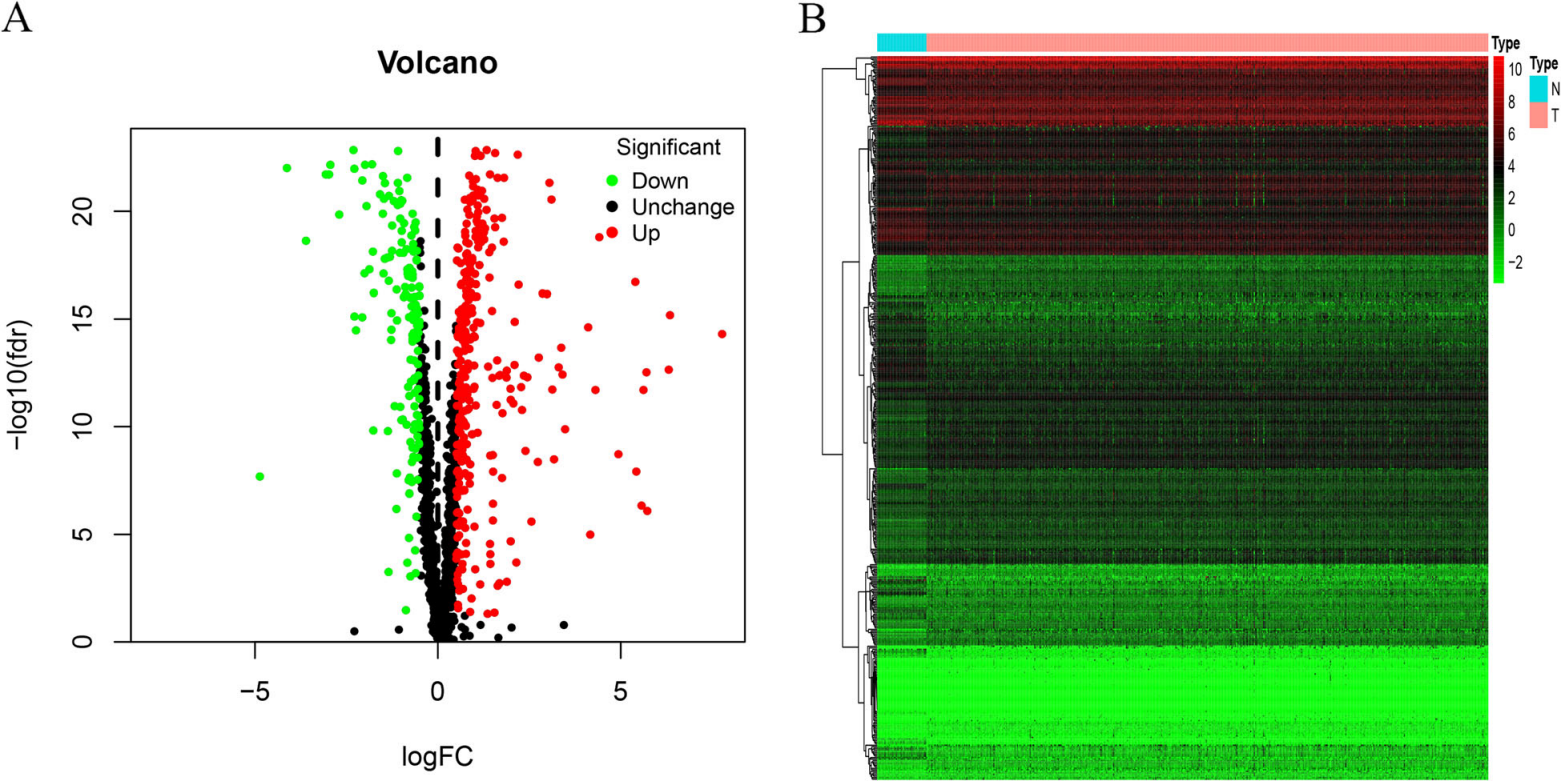
Volcano and heat map of RNA-binding protein DEGs. **a** Volcano map. **b** Heat map. Red nodes represent upregulated genes, and green nodes represent downregulated genes.

Transcriptome sequencing data of 1493 RBPs of CRC was obtained from the TCGA database. The differential expression analysis was conducted to find out that there were 314 upregulated genes and 155 downregulated genes, based on which volcano and heat maps were drawn as shown in Fig. 2.

### Functional enrichment analyses of DEGs

**Fig. 3:**
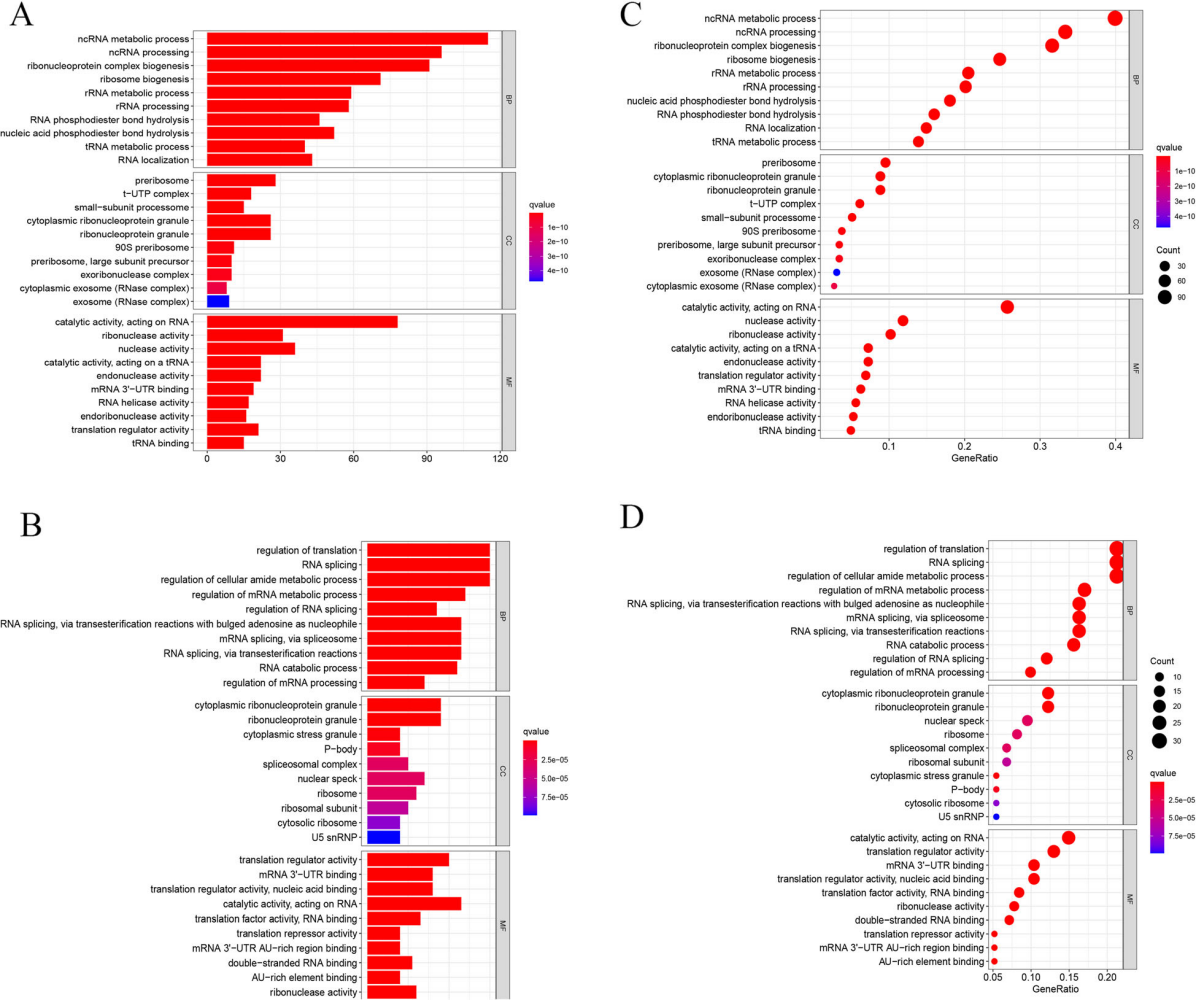
The Gene Ontology analyses of 469 RNA-binding protein DEGs. **a** Barplot shows GO functional enrichment analysis predicted upregulated DEGs, including biological process, cellular components and molecular functions. The colour indicates the significance of the *p* value. **b** Bubble shows GO functional enrichment analysis predicted upregulated DEGs. The size of the circle represents the number of genes enriched in the entry, and the colour indicates the significance of the *p* value. **c** Barplot shows GO functional enrichment analysis predicted downregulated DEGs. **d** Bubble shows GO functional enrichment analysis predicted downregulated DEGs.

**Fig. 4:**
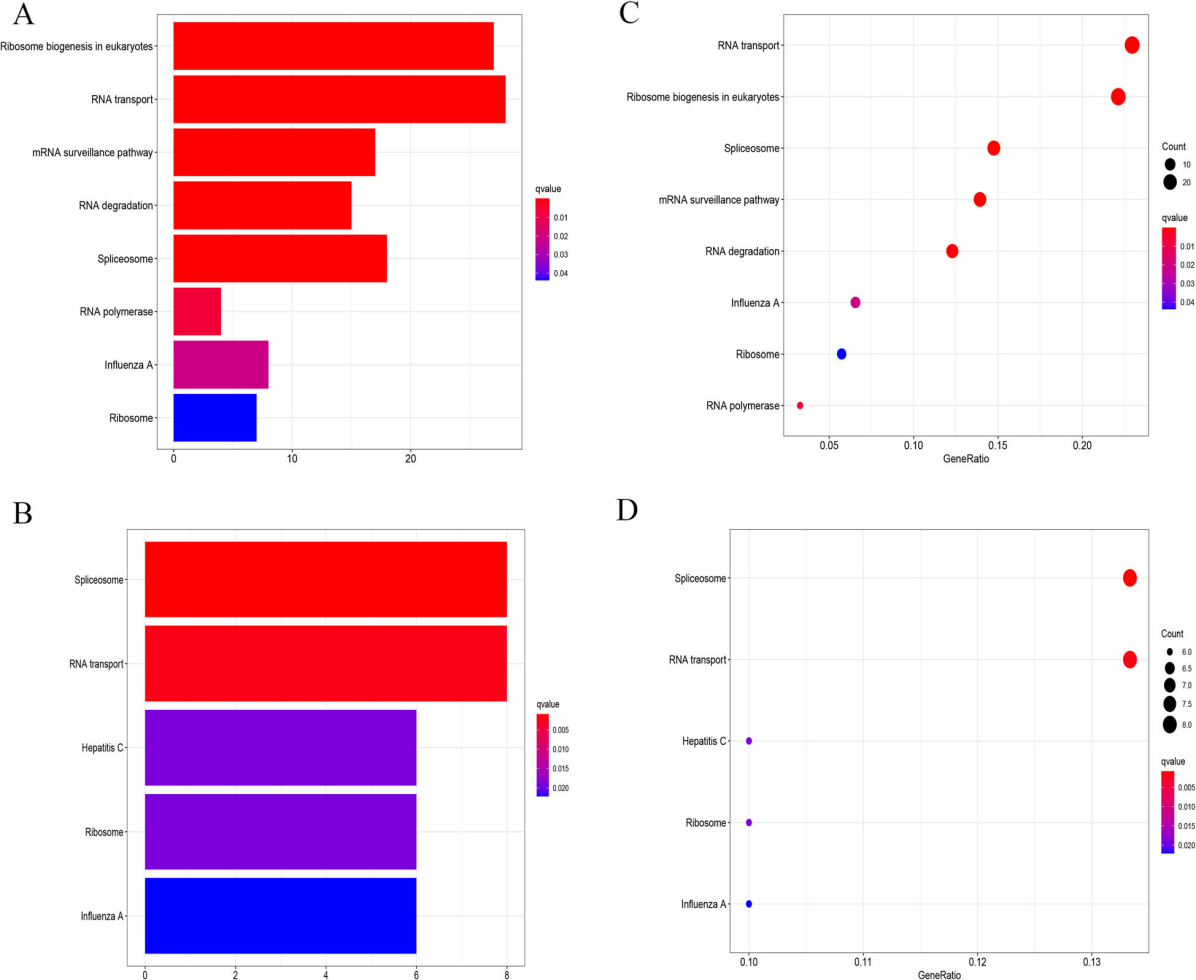
The KEGG pathway enrichment analyses of 469 RNA-binding protein DEGs. **a** Barplot shows KEGG pathway analysis predicted upregulated DEGs. The colour indicates the significance of the *p* value. **b** Bubble shows KEGG pathway analysis predicted upregulated DEGs. The size of the circle represents the number of genes enriched in the entry, and the colour indicates the significance of the *p* value. **c** Barplot shows KEGG pathway analysis predicted downregulated DEGs. **d** Bubble shows KEGG pathway analysis predicted downregulated DEGs

The up- and downregulated genes of DEGS were analysed for GO function and KEGG pathway enrichment, while both barplot and bubble were plotted. The enriched GO terms were divided into CC, BP and MF ontologies. The top 10 most relevant items were selected, as shown in Fig. 3. With regard to the upregulated genome, the results of GO analysis indicated that DEGs were mainly enriched in BPs, including ncRNA metabolic process, ncRNA processing, ribonucleoprotein complex biogenesis and ribosome biogenesis and so on. CC analysis revealed that the DEGs were significantly enriched in preribosome, t-UTP complex, small-subunit processome and cytoplasmic ribonucleoprotein granule and so on. As for the MF, the DEGs were enriched in catalytic activity, thus influencing RNA and ribonuclease activity. In the downregulated genome, BP analysis demonstrated that the DEGs were significantly enriched, as reflected in the regulation of translation, RNA splicing, the regulation of cellular amide metabolic process and so on. CC analysis showed that the DEGs were significantly enriched in cytoplasmic ribonucleoprotein granule, ribonucleoprotein granule, cytoplasmic stress granule, etc. As for the MF, the DEGs were enriched in translation regulator activity, mRNA 3′-UTR binding and so on. Regarding the results of KEGG pathway analysis as shown in Fig. 4, the DEGs in the upregulated genome were primarily enriched in the pathways in Ribosome biogenesis in eukaryotes and RNA transport, etc. In the downregulated genome, the DEGs were largely enriched in the pathways in Spliceosome and RNA transport, etc.

### PPI network construction

**Fig. 5:**
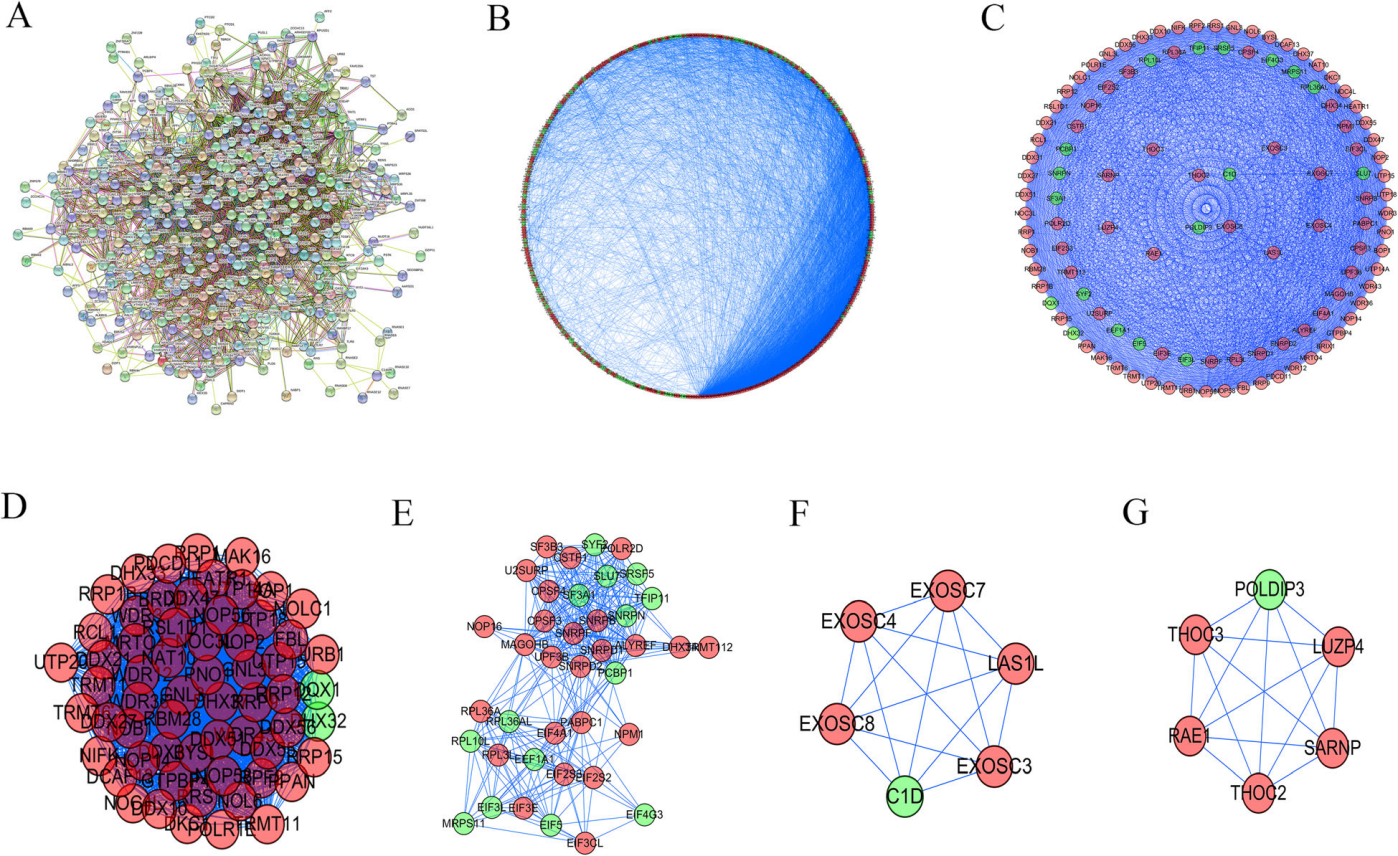
RNA-binding proteins DEGs are used to construct protein-protein interaction networks and subnetworks. **a** PPI interaction network map obtained from STRING website. **b** Cytoscape visualizes the genes of the interacting PPI network. Red nodes represent upregulated genes, while blue nodes refer to downregulated genes. **c** Four MCODE modules visualization. **d**–**g** Four most significant MCODE components form the PPI network

The protein interactions among the DEGs were predicted using STRING tools. A total of 442 nodes and 6233 edges in the PPI network were obtained, as shown in Fig. 5a. Then, Cytoscape software was applied to draw a network diagram of 442 genes, as shown in Fig. 5b. Besides, four key sub-networks with the MCODE plug-in were extracted. GO was performed (Table 1) and KEGG enrichment analysis was conducted (Table 2) on the genes of the four sub-networks, respectively. Finally, the four sub-networks were visualized, as shown in Fig. 5c–e. The number of hub genes in these 4 sub-networks is 61, 39, 6 and 6, respectively.

**Table 1:** The GO function enrichment analysis of four most significant MCODE components

Ontology	ID	Description	Count	*p* value	*p*.adjust
Sub-network 1					
BP	GO:0042254	Ribosome biogenesis	46	1.57E−74	5.06E−72
BP	GO:0016072	rRNA metabolic process	43	7.30E−73	1.18E−70
BP	GO:0006364	rRNA processing	42	2.21E−71	2.38E−69
CC	GO:0030684	Preribosome	27	5.03E−51	1.71E−49
CC	GO:0034455	t-UTP complex	18	2.51E−33	4.26E−32
CC	GO:0032040	Small-subunit processome	15	5.16E−29	5.84E−28
MF	GO:0140098	Catalytic activity, acting on RNA	20	8.42E−19	6.90E−17
MF	GO:0003724	RNA helicase activity	12	2.62E−17	1.08E−15
MF	GO:0030515	snoRNA binding	8	1.60E−14	4.38E−13
Sub-network 2					
BP	GO:0000377	RNA splicing, via transesterification reactions with bulged adenosine as nucleophile	21	7.37E−26	1.03E−23
BP	GO:0000398	mRNA splicing, via spliceosome	21	7.37E−26	1.03E−23
BP	GO:0000375	RNA splicing, via transesterification reactions	21	8.71E−26	1.03E−23
CC	GO:0071013	Catalytic step 2 spliceosome	13	6.97E−22	6.62E−20
CC	GO:0000974	Prp19 complex	13	2.03E−21	9.66E−20
CC	GO:0005682	U5 snRNP	13	3.10E−19	9.83E−18
MF	GO:0090079	Translation regulator activity, nucleic acid binding	10	2.82E−14	2.23E−12
MF	GO:0003743	Translation initiation factor activity	8	1.54E−13	4.37E−12
MF	GO:0008135	Translation factor activity, RNA binding	9	1.66E−13	4.37E−12
Sub-network 3					
BP	GO:0000460	Maturation of 5.8S rRNA	6	5.04E−18	5.24E−16
BP	GO:0034427	Nuclear-transcribed mRNA catabolic process, exonucleolytic, 3′-5′	4	6.22E−13	3.23E−11
BP	GO:0043629	ncRNA polyadenylation	4	1.47E−12	3.67E−11
CC	GO:1905354	Exoribonuclease complex	6	2.17E−18	3.69E−17
CC	GO:0000176	Nuclear exosome (RNase complex)	5	1.50E−15	1.27E−14
CC	GO:0000178	Exosome (RNase complex)	5	1.03E−14	5.82E−14
MF	GO:0017091	AU-rich element binding	3	7.07E−08	8.18E−07
MF	GO:0000175	3′-5′-Exoribonuclease activity	3	1.29E−07	8.18E−07
MF	GO:0016896	Exoribonuclease activity, producing 5′-phosphomonoesters	3	1.54E−07	8.18E−07
Sub-network 4					
BP	GO:0051028	mRNA transport	6	2.64E−13	1.90E−11
BP	GO:0050657	Nucleic acid transport	6	1.13E−12	2.23E−11
BP	GO:0050658	RNA transport	6	1.13E−12	2.23E−11
CC	GO:0000346	Transcription export complex	3	5.69E−09	9.11E−08
CC	GO:0016607	Nuclear speck	4	2.35E−06	1.88E−05
CC	GO:0000784	Nuclear chromosome, telomeric region	2	0.000588204	0.003137089

**Table 2:** The KEGG function enrichment analysis of four most significant MCODE components

List	ID	Description	Count	*p* value	*p*.adjust
Sub-network1	hsa03008	Ribosome biogenesis in eukaryotes	19	1.84E−32	3.68E−32
Sub-network2	hsa03040	Spliceosome	13	4.08E−13	2.85E−12
	hsa03013	RNA transport	12	1.00E−10	3.51E−10
	hsa03015	mRNA surveillance pathway	7	5.47E−07	1.28E−06
	hsa03010	Ribosome	5	0.001767902	0.003093829
Sub-network3	hsa03018	RNA degradation	5	4.75E−10	4.75E−10

### Construction and analysis of prognostic models

**Fig. 6:**
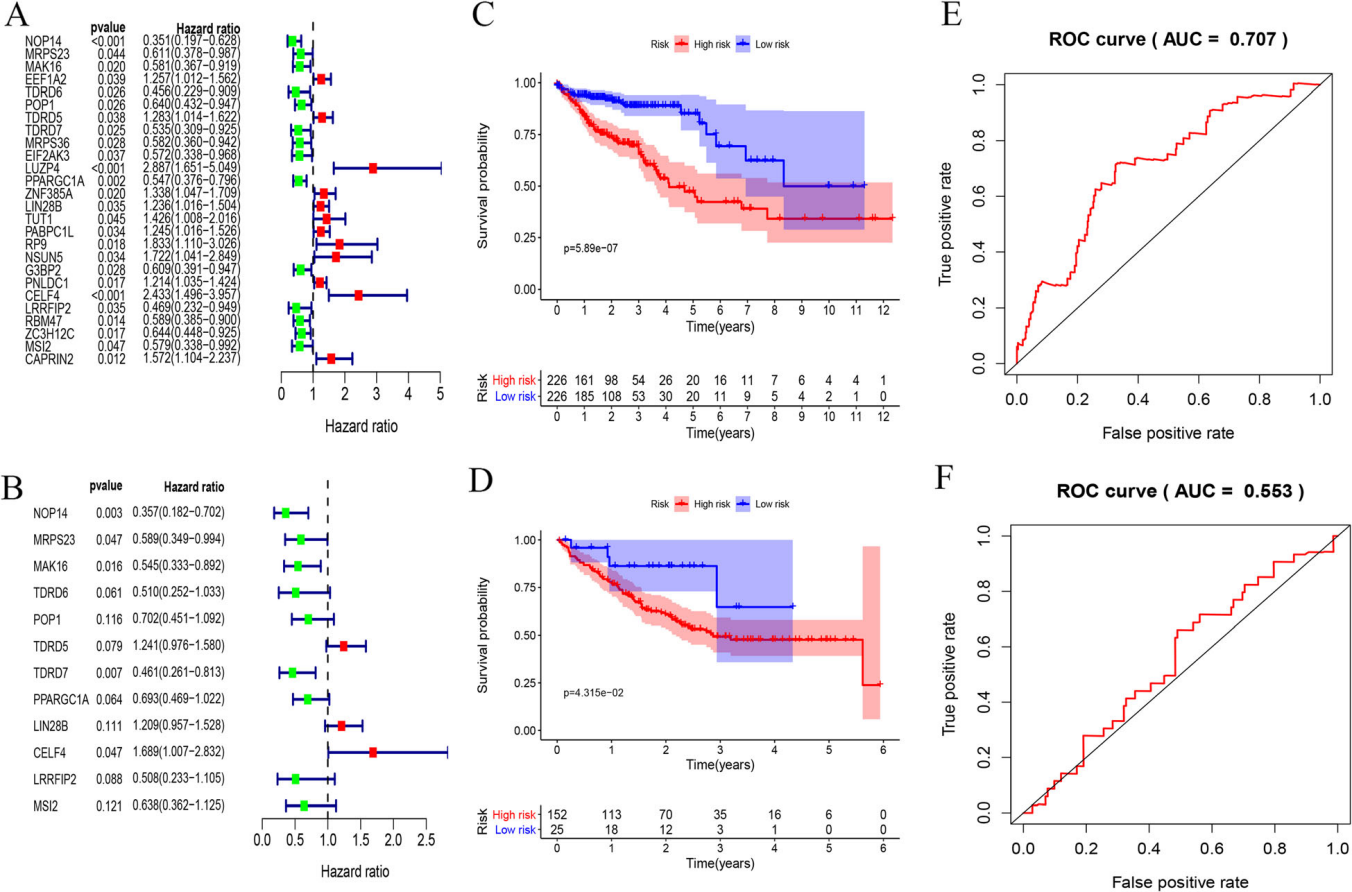
RNA-binding protein DGEs are used to construct prognostic models, survival analysis and verification of GEO data sets. **a** The 19 prognostic-related RBPs shown in the forest map, red indicates high-risk genes and green denotes low-risk genes. **b** The 12 RBPs obtained by constructing the prognostic model shown in the forest map. **c** Survival analysis curve of training set, red indicates patients in the high-risk group, blue denotes patients in the low-risk group. **d** Survival analysis curve of test set. **e** ROC curve of training set. **f** ROC curve of test set

**Fig. 7:**
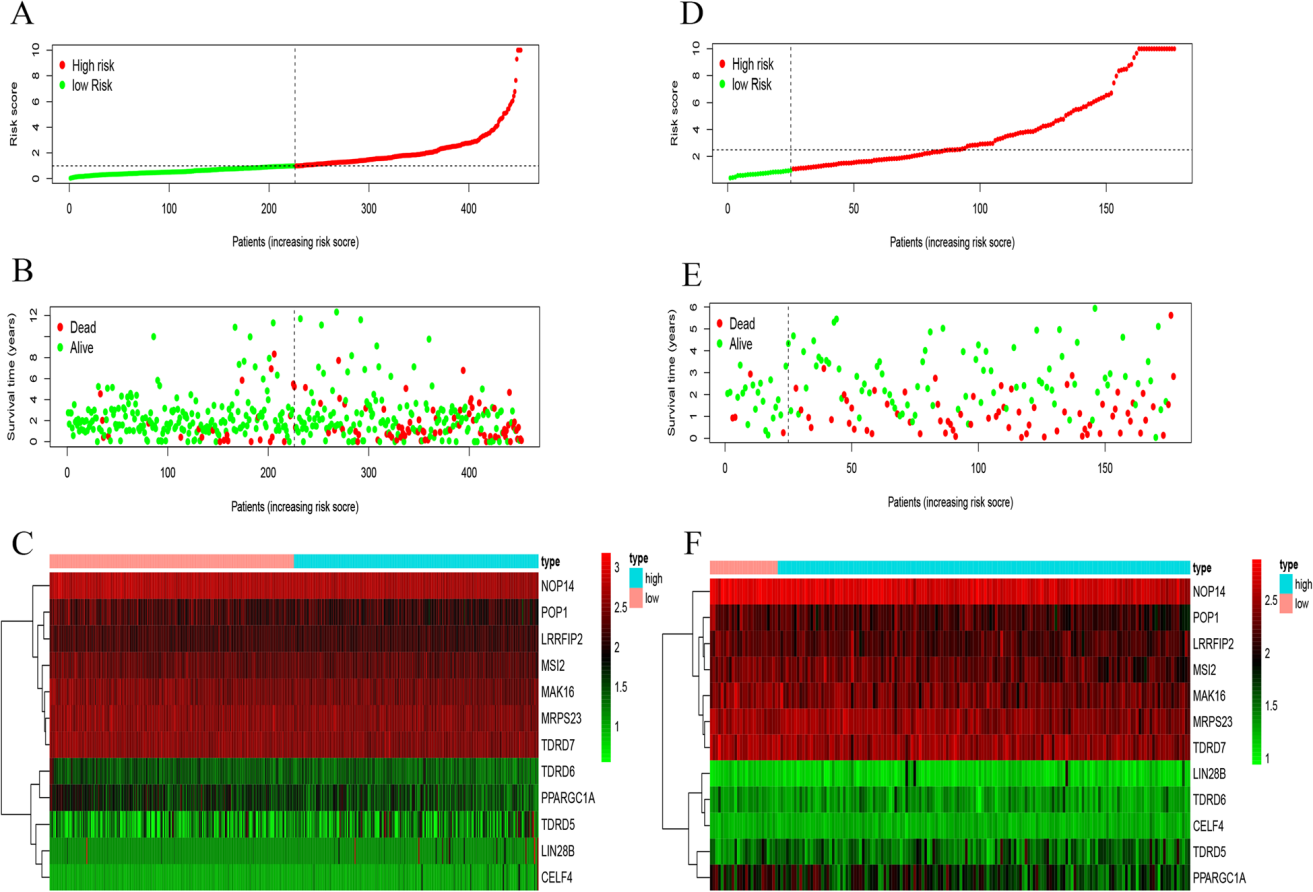
Risk curve of training and test sets. **a** The risk score distribution of training set. **b** The distribution of survival status for training set. **c** In training set, the heat map of 12 RBPs for the high- and low-risk groups. **d** The risk score distribution of test set. **e** The survival status distribution for test set. **f** In test set, the heat map of 12 RBPs for the high- and low-risk groups

Cox regression analysis was carried out of the prognostic value of 442 RBPs interacting with survival time, 19 RBPs related to survival were screened and a forest map was drawn as shown in Fig. 6a. Then, a prognostic model was constructed for the RBPs related to prognosis, and a prognostic marker gene comprised of 12 RBPs was established. These twelve genes are nucleolar protein 14 (NOP14), mitochondrial ribosomal protein S23 (MRPS23), MAK16 homolog (MAK16), tudor domain-containing 6 (TDRD6), processing of precursor 1 (POP1), tudor domain-containing 5 (TDRD5), tudor domain-containing 7 (TDRD7), peroxisome proliferator-activated receptor gamma coactivator 1-alpha (PPARGC1A), lin-28 homolog B (LIN28B), CUGBP Elav-like family member 4 (CELF4), leucine-rich repeat flightless-interacting protein 2 (LRRFIP2) and Musashi RNA-binding protein 2 (MSI2). Then, the corresponding forest map was drawn for these twelve genes as shown in Fig. 6b. Among them, TDRD5, ELF4 and LRRFIP2 are classed as high-risk genes, while the rest is classed as low-risk genes. Based on the established model, the risk value of each patient was calculated. According to the median value, the patients in the training set and the test set were divided into either a high-risk group or a low-risk group. Among them, the number of patients in the training set as well as the high-risk group was 226. The number of patients in the low-risk group was 226. In the test set, the number of patients in the high-risk group was 152 and that of patients in the low-risk group was 25. According to the results, the patients with high-risk scores had a shorter survival time, as shown in Fig. 6c, d. Finally, in terms of survival prediction, the ROC curve showed a relatively decent performance, as shown in Fig. 6e, f. The AUC value in the training set was 0.754 and the AUC value in the test set was 0.553. Then, the risk curves were plotted for the training and test sets, as shown in Fig. 7, which reveals that their abscissas are the same. They were divided into high and low-risk groups by the median value. The patients were ranked by risk value in ascending order. The risk value of patients from left to right increased on a continued basis, as did the risk of fatality.

**Fig. 8:**
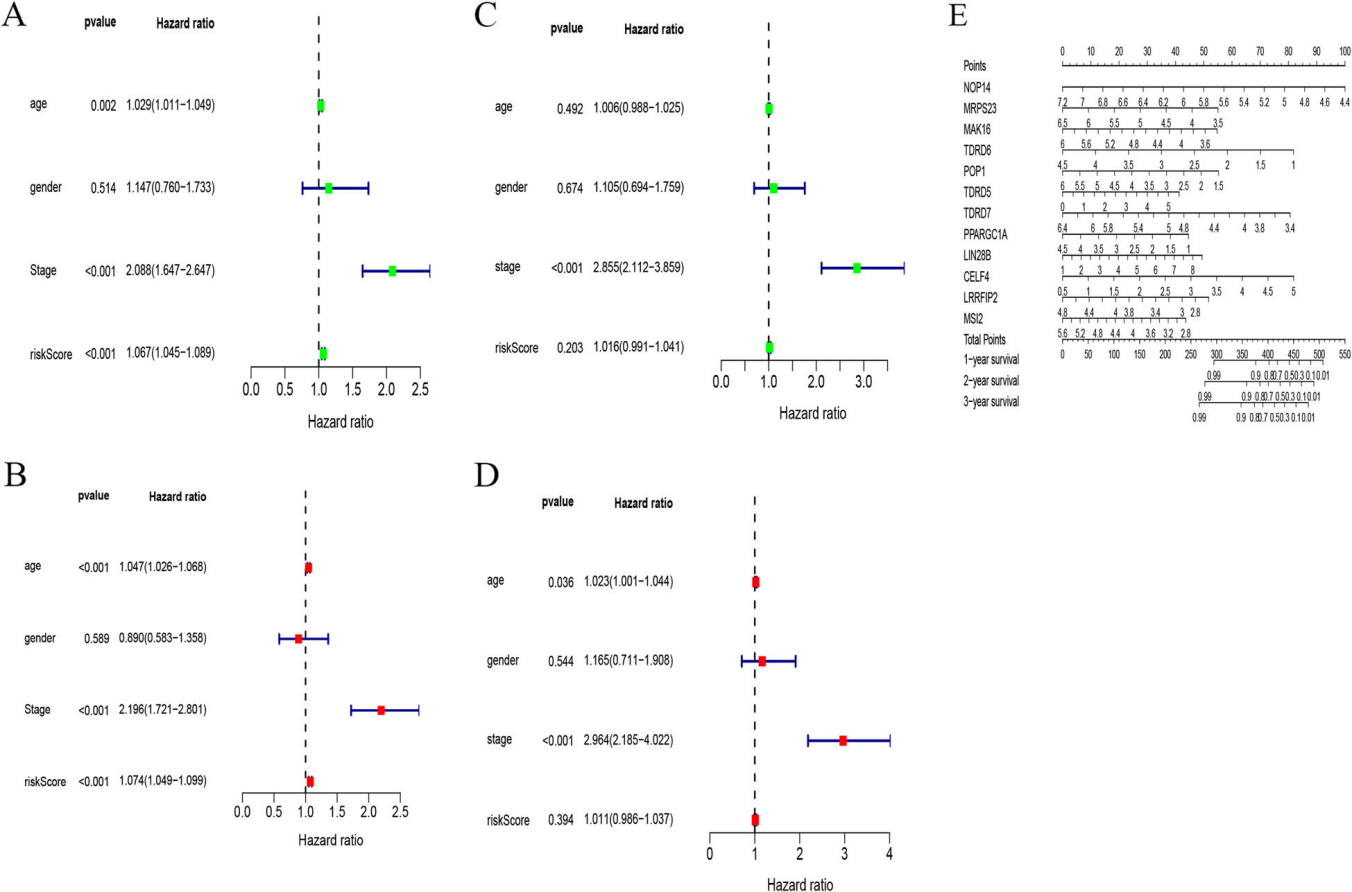
Independent prognosis analysis and prediction of 1, 2 and 3 years of nomograms of CRC patients in the training and test sets. **a** Single-factor prognosis analysis of training set. **b** Multi-factor prognosis analysis of training set. **c** Single-factor prognosis analysis of test set. **d** Multi-factor prognostic analysis of test set. **e** The nomograms for predicting 1-year, 2-year and 3-year survival probability of patients with CRC for training set

Then, independent prognostic analysis was conducted of univariate and multivariate for the training and test sets, as shown in Fig. 8a–d. According to the results of single-factor independent prognosis analysis, for the training and test sets, age and tumour stage can be treated as independent prognostic factor for the survival of colorectal patients (*p* < 0.05). In the multivariate independent prognostic analysis, age and stage can be taken as independent prognostic factor for CRC in the test set (*p* < 0.05). For the training set, however, only stage can be taken as independent prognostic factors for CRC (*p* < 0.01), not age (*p* = 0.492).

Finally, nomograms were plotted for these 12 RBP prognostic genes in the training set to predict the survival time of the patients, as shown in Fig. 8e. The RNA expression of 12 RBPs was applied as parameters to draw the point line in nomograms. The scores were added to obtain the total score, which can be used to predict the 1-year, 2-year and 3-year survival rates among CRC patients.

## Discussion

As one of the most common malignant tumours, CRC is characterized by a high recurrence rate and poor prognosis, especially in developed countries. It is the third most common cancer among males and ranks second among females [9, 10]. To date, various methods have been applied to predict biomarkers of CRC prognosis [11]. RBPs can regulate mRNA stability and contribute to cancer-associated pathways [12]. In this paper, the RBPs of CRC were analysed. Through a series of analyses, 12 marker genes related to the prognosis of CRC were identified.

Tudor domain-containing (TDRD) refers to a family of evolutionarily conserved proteins. In general, PIWI and TDRD proteins are recognized as the major influencing factors in piRNA biogenesis and the development of germ cells [13]. In a previous study, it was found that methyl lysine-bound TDRDs are primarily involved in histone modification and chromatin remodelling, while methyl arginine-bound TDRDs are usually associated with RNA metabolism, alternative splicing, small RNA pathways and germ cell development [14, 15]. TDRDs have now been detected in various cancers. TDRD9 is highly expressed in a subset of non-small cell lung carcinomas and derived cell lines through hypomethylation of its CpG island [16]. TDRD1 is closely associated with ERG overexpression in primary prostate cancer [17]. According to the findings by Jiang et al. [18], 7 TDRD genes (PHF20L1, ARIB4B, SETDB1, LBR, TDRKH, TDRD10 and TDRD5) showed high levels of amplification in more than 10% of TCGA breast cancer datasets. TDRD5 has significant prognostic value for hepatocellular carcinoma (HCC). Patients with higher TDRD5 expression exhibit significantly poorer overall survival than patients with low TDRD5 expression [19]. An early study revealed that TDRD5 was expressed in normal gastric and colonic mucosal tissues, suggesting the possibility that the TDRD5 gene is modified in CRC [20]. TDRD6 is capable of differentiating irradiated prostate cancer patients into early and late relapse groups [21]. In addition, TDRD7 may play a certain role in the migration of tumour cells [22]. In an analysis of CRC, Mo et al. [23] discovered not only frameshift mutations but also intratumoural heterogeneity of TDRD1, TDRD5 and TDRD9, which in combination might alter TDRD gene functions and affect the tumorigenesis of high microsatellite instability CRC. In our study, it was found that TDRD5, TDRD6 and TDRD7 are differentially expressed in CRC, and further studies on the role of these three genes in colon cancer are needed.

POP1 is a component of ribonuclease P, which is a ribonucleoprotein complex that generates mature tRNA molecules by cleaving their 5′ end s[24, 25]. In addition, it is a component of the MRP ribonuclease complex, which cleaves pre-rRNA sequences [26]. In a previous study, POP1 was found to be enriched in human prostate cancer cell lines [27], suggesting that it may be suitable as a potential marker for the diagnosis and prognosis of prostate cancer. In addition, POP1 is upregulated in CRC and applicable as a prognostic factor for CRC. Nevertheless, there is still no relevant research on the mechanism of POP1 in CRC, so further studies are necessary.

PPARGC1A, also known as PGC1α, is a transcriptional coactivator of genes encoding proteins responsible for the regulation of mitochondrial biogenesis and function [28]. D’Errico et al. [29] discovered that in the presence of Bax, PGC1α-induced ROS accumulation is one of the main apoptosis-driving factors in CRC cells. They also found that PGC1α induced mitochondrial proliferation and activation in human intestinal cancer cells [30]. Shin et al. [31] demonstrated that PGC1α overexpression was effective in upregulating the proliferation of HEK293 and CT26 cells. In addition, its overexpression was correlated with an enhancement of tumourigenesis. In a case-control study, heterozygous carriers of rs3774921 in PGC1α showed an increased risk of CRC [32]. PGC1α plays an essential role in the pathogenesis of colon cancer. In a clinical study, the expression of PGC1α was assessed in 17 CRC patients using real-time quantitative PCR, and the mRNA level of PGC1α was found to be decreased in the tumours of most patients [33]. However, immunohistochemistry has also been performed to detect the expression of PGC1α. The results revealed that 51.9% of the 108 CRC samples were positive, while no or weak PGC1α expression was detected in the nuclei of normal mucosa cells. PGC1α expression is demonstrated to be related to lymph node metastasis. Thus, it can serve as a possible prognostic marker [34]. Our results also show that PGC1α can be used as an independent prognostic factor for CRC.

It is thought that LRRFIP2 functions as an activator of the canonical Wnt signalling pathway, which is associated with DVL3, a factor upstream of CTNNB1/beta-catenin. It positively regulates Toll-like receptor (TLR) signalling in response to agonists, probably by competing with the negative FLII regulator for MYD88 binding, which plays a crucial role in the progression of colon cancer [35, 36]. In this study, LRRFIP2 was identified as a candidate gene for alternative splicing in colon and prostate cancer. There were three splice variants that differed in their inclusion or skipping of exons 5 and/or 6. These exons contain five predicted putative serine phosphorylation sites and one putative O-glycosylation site and could modulate LRRFIP2 protein function [37]. As a familial hereditary disease, hereditary nonpolyposis CRC (Lynch syndrome) is mainly caused by DNA mismatch (mismatch repair). In Lynch syndrome, Morak and colleagues discovered a paracentric inversion on chromosome 3p22.2 between the DNA mismatch repair gene MLH1 and the downstream LRRFIP2 gene transcribed in the antisense direction. This generates two new stable fusion transcripts, thus removing the MLH1 gene and protein function [38]. In another study conducted on a Lynch syndrome family, it was found that the MLH1.ITGA9 fusion allele caused loss of heterozygosity (LOH) in five genes, including LRRFIP2, which resulted in the loss of mismatch repair capabilities [39]. Thus, LRRFIP2 may play a critical role in the pathogenesis of CRC.

CELF4 is responsible for encoding a protein with three domains that bind an RNA recognition motif and regulate pre-mRNA alternative splicing. Some studies showed that CELF4 was hypermethylated in endometrial cancer. Methylated CELF4 may be suitable for endometrial cancer screening of cervical smears [40]. Further research is still needed to determine the role of CELF4 in tumours.

As a member of the Musashi family, MSI2 belongs to the family of *Drosophila melanogaster* RNA-binding proteins. It has been identified as a critical regulator of haematopoietic stem cell (HSC) self-renewal and fate determination [41, 42]. In this study, MSI2 was found to be a central component in an unknown oncogenic pathway to promote intestinal transformation via the PDK-AKT-mTORC1 axis [43]. MSI2 is highly expressed in a variety of cancers, including HCC and lung cancer [44, 45]. Recent studies on colon cancer cell lines have suggested that both USP10 and MSI2 proteins are upregulated. In addition, ubiquitin-specific protease 10 (USP10) could stabilize the oncogenic factor MSI2 through deubiquitination [46]. The expression of MSI2 was detected in CRC and control specimens from 164 patients by the tissue microarray technique and immunohistochemical staining. MSI2 was highly expressed in 32.9% (54/164) of CRC samples. In addition, high MSI2 expression was related to liver metastasis in CRC patients [47]. In other cancers, Guo et al. found that MSI2 expression was markedly increased in both pancreatic ductal adenocarcinoma (PDAC) cell lines and human PDAC specimens, and high MSI2 expression was associated with poor prognosis of PDAC [48]. High expression of MSI2 mRNA is associated with decreased survival in acute myeloid leukaemia [49]. Furthermore, MSI2 may act as a prognostic biomarker in patients with cervical cancer [50], bladder cancer [51] and oesophageal squamous cell carcinoma [52]. It was also found that its expression is upregulated in CRC, which makes it applicable as a prognostic marker gene for CRC.

LIN28, an oncofoetal RNA-binding protein, modulates stem cell maintenance, somatic reprogramming, metabolism, organismal growth, tissue development and tumourigenesis [53]. Two paralogues of LIN28 were included, LIN28A and LIN28B. It is well established that LIN28A and LIN28B inhibit let-7 family miRNAs and derepress let-7 targets, including Ras, PI3K/AKT, Myc, Hmga2 and Igf2bps, thus promoting oncogenesis [54, 55]. In liver cancer stem cells, Fang et al. found that overexpression of MSI2 resulted in the upregulation of LIN28A. Stemness and chemotherapeutic drug resistance induced by MSI2 overexpression were dramatically reduced by LIN28A knockdown. Moreover, MSI2 and LIN28A levels positively correlated with the clinical severity and prognosis in HCC patients [56]. King et al. [57] found that LIN28B overexpression is associated with reduced survival time and increased probability of tumour recurrence in patients. Constitutive LIN28B expression promotes not only tumorigenesis but also LGR5 and PROM1 expression in colonic epithelial cells [58]. In addition, LIN28B promotes the proliferation, colony formation and tumourigenesis of colon cancer cells by increasing BCL-2 expression [59]. A clinical study found that LIN28A and LIN28B were overexpressed in oesophageal cancer cells, especially on the invasive front. High expression of LIN28A and LIN28B correlated significantly with lymph node metastasis and poor prognosis [60]. Hu et al. found that gastric adenocarcinoma (GAC) patient survival time was negatively correlated with the LIN28B expression level, whereby higher LIN28B expression correlated with shorter survival time [61]. In PDAC patients, high LIN28B expression was significantly correlated with high levels of lymphatic metastasis, distant metastasis and a poor prognosis. In addition, patients with increased LIN28B had markedly reduced overall survival compared to those with low LIN28B in HCC [62] and oral squamous cell carcinoma (OSCC) [63]. Thus, LIN28B is highly expressed in CRC and plays an important role in its pathogenesis, indicating that it is suitable as a target gene for CRC prognosis.

NOP14 is a stress-responsive gene required for 18S rRNA maturation and 40S ribosome production [64]. As indicated by Zhou et al. [65], NOP14 in pancreatic cancer cells promotes motility, proliferation and metastatic capacity. According to the findings by Du et al. [66], NOP14 induced tumour invasion and metastasis by improving the stability of mutp53 mRNA. By inhibiting the Wnt/β-catenin pathways, NOP14 suppresses breast cancer [67]. In addition, NOP14 can reduce melanoma cell proliferation and metastasis by regulating the Wnt/b-catenin signalling pathway [68]. In clinical studies of patients with ovarian cancer, downregulation of NOP14 was associated with a significantly worse survival rate [69]. This study showed that the expression of NOP14 was upregulated in CRC, but its role in pathogenesis requires further research and confirmation.

The MRPS23 gene, which is responsible for encoding a 28S subunit protein, has been found to be overexpressed in breast cancer [70], uterine cervical cancer [71], HCC [72], colorectal cancer [73] and uterine leiomyoma [74]. As revealed by Gao et al. [75], inhibiting MRPS23 could lead to a significant reduction in breast cancer metastasis by inhibiting the EMT phenotype. Pu et al. found that high MRPS23 levels can predict poor clinical outcomes in HCC [72]. Although the expression of MRPS23 is increased in CRC, its specific pathogenesis remains unclear.

MAK16 encodes a ribosomal protein and plays an important role in ribosome biogenesis throughout the cell cycle [76]. In this study, it was found that mutations in MAK16 can induce cell cycle arrest at G1 phase, during which the cell synthesizes mRNA and proteins in preparation for cell division [77]. At present, there is still no study of the role of MAK16 in the pathogenesis of tumours, which requires further research to confirm.

In this paper, a discussion was conducted about the role of the 12 identified genes in tumours. Although some genes were found irrelevant to the pathogenesis of CRC, their biological functions and changes in their expression in CRC suggest that they may play a role in CRC to some extent, and further experiments need to be conducted for verification. This is also a limitation of our study. More research is needed to explore the pathogenesis of CRC.

The above genes are related to the prognosis of CRC. More research, especially experimental studies, is needed to verify the specific function of each gene. Our findings may improve the understanding of the incidence and prognosis of CRC, thus providing a reference for further improvement of the diagnosis and treatment of CRC.

## Conclusions

In summary, 12 prognostic RBPs were obtained through TCGA database analysis, including NOP14, MRPS23, MAK16, TDRD6, POP1, TDRD5, TDRD7, PPARGC1A, LIN28B, CELF4, LRRFIP2 and MSI2, which were then verified through the sample data obtained from the GEO database. In CRC, NOP14, MRPS23, MAK16, TDRD6, POP1, TDRD5, LIN28B and MSI2 were upregulated, while TDRD7, PPARGC1A, CELF4 and LRRFIP2 were downregulated. These genes are related to the prognosis of CRC. More research is deemed necessary to verify the specific function of each gene, especially experimental studies. Our findings may improve the understanding of the incidence and prognosis of CRC, thus providing reference for the further exploration of the diagnosis and treatment of CRC.

## Data Availability

The datasets supporting the conclusion of this article are included within the article.

## Abbreviations

RBPs: RNA-binding proteins
CRC: Colorectal cancer
DEGs: Differentially expressed genes
GO: Gene Ontology
KEGG: Kyoto Encyclopedia of Genes and Genomes
MBNL1: Muscleblind-like 1
EMT: Epithelial-mesenchymal transition
BP: Biological processes
MF: Molecular functions
CC: Cellular components
NOP14: Nucleolar protein 14
MRPS23: Mitochondrial ribosomal protein S23
MAK16: MAK16 homolog
TDRD6: Tudor domain-containing 6
POP1: Processing of precursor 1
TDRD5: Tudor domain-containing 5
TDRD7: Tudor domain-containing 7
PPARGC1A: Peroxisome proliferator-activated receptor gamma coactivator 1-alpha
LIN28B: Lin-28 homolog B
CELF4: CUGBP Elav-like family member 4
LRRFIP2: Leucine-rich repeat flightless-interacting protein 2
MSI2: Musashi RNA-binding protein 2
TDRD: Tudor domain-containing
HSC: Haematopoietic stem cell.
